# Cellular MicroRNA Let-7a Suppresses KSHV Replication through Targeting MAP4K4 Signaling Pathways

**DOI:** 10.1371/journal.pone.0132148

**Published:** 2015-07-21

**Authors:** Xiaohua Tan, Yuan Gao, Yulong Nan, Jinxia Zhang, Chunhong Di, Xiaobo Wang, Fuzhi Lian, Yifei Cao, Yu Hu, Liangwen Xu, Haiyan Ma, Yu Hong, Tingjie Liu, Yinyin Wu, Xianrong Xu, Yutao Yan, Lei Yang

**Affiliations:** School of Medicine, Hangzhou Normal University, Hangzhou, Zhejiang, China; University of Southern California Keck School of Medicine, UNITED STATES

## Abstract

**Background:**

Kaposi’s sarcoma (KS)-associated herpesvirus (KSHV) is the etiologic agent of KS, the most common AIDS-related malignancy. The majority of KS tumor cells harbor latent KSHV virus but only a small percentage undergoes spontaneous lytic replication. Viral reactivation from latency is crucial for the pathogenesis and development of KS, but the cellular mechanisms underlying the switch between viral latency and replication are not well understood.

**Methods:**

The level of let-7 miRNAs and MAP4K4 in KSHV infected 293T cells were quantified by real-time PCRs. Let-7 expression was silenced by the miRNA sponge technique. In let-7a transfected 293T cells, the expression of MAP4K4 was measured by real-time PCR and western blot. Luciferease expression was employed to examine the effect of let-7a on the 3’-untranslated region (UTR) of the *MAP4K4* gene in 293T cells. Real-time PCR was used to quantify the KSHV copy numbers in BC-3 cells in which the expression of let-7a and/or MAP4K4 were altered. Finally, ERK, JNK and p38 protein production and their phosphorylation status were detected by western blots in let-7a or MAP4K4 transfected BCBL-1 cells.

**Results:**

The expression of microRNA let-7 was dramatically decreased in KSHV infected 293T cells, but that of MAP4K4 was increased significantly. Let-7a is physically associated with and targets the MAP4K4 3’UTR, and inhibits MAP4K4 expression at both mRNA and protein levels. MAP4K4 stimulates KSHV reactivation from latency, whereas let-7a inhibits the function of MAP4K4 by reversing the function of MAP4K4 on JNK, phospho-JNK and phospho-ERK1/2 levels.

**Conclusion:**

Our results establish that let-7a specifically suppresses MAP4K4 expression, and further inhibits KSHV reactivation by interfering with the function of MAP4K4 on the MAPK pathway, highlighting let-7a as a potential treatment for KS.

## Introduction

Kaposi’s sarcoma-associated herpesvirus (KSHV), or human herpesvirus 8 (HHV-8), is the etiologic agent of a number of human malignancies, including Kaposi’s sarcoma (KS)[1], primary effusion lymphoma (PEL)[2], and multicentric Castleman disease (MCD) [3]. Like many other human herpesviruses, KSHV remains latent in the majority of infected cells. In a small proportion of those cells, however, KSHV can undergo spontaneous lytic replication (called reactivation) at any given time [4–5], but the underlying mechanisms involved in the switch between latency and replication are not clearly understood. Importantly, it is this low percentage of viral reactivation that is believed to play an important role in viral persistence, tumorigenesis [6], angiogenesis, and proliferation of neighboring cells and onset of inflammation [7–8].

MicroRNAs (miRNA) are endogenous, small noncoding RNAs, with a length of approximately 19–24 nucleotides, playing important roles in the negative regulation of gene expression by base pairing to complementary sites on the 3’-untranslated region (3’-UTR) of target messenger RNAs(mRNAs) [9], thus triggering mRNA degradation or blocking its translation [10–12]. It is estimated that miRNAs are responsible for the regulation of up to 30% of the gene expression in humans [13] and orchestrate fundamental roles in a wide variety of biological processes, including development, apoptosis, proliferation, differentiation, immune response, cell cycle control, energy metabolism, and many pathological conditions such as cancer [14–15] Differential miRNA expression has already been regarded widely as a hallmark of tumorigenesis, since most miRNAs are downregulated in cancer cells, which in turn promotes further tumorigenesis. Thus, low miRNA represents a common defect in cancer cells that has yet to be exploited therapeutically.

Increasing evidence suggests that viral infection can influence an expression profile of host miRNAs, either by defensive host signaling against viral infection or by viral hijacking to favor infection. Conversely, it was pointed out that host miRNAs are also important in regulating viral miRNAs and balancing virus-host interactions [16–18]. Recently, it was found that miRNAs, including the let-7 family and miR-220/221, are downregulated in KSHV-associated cancers, such as PEL and KS [19]. Cellular miRNAs 498 and 320d regulate KSHV lytic replication[20]. Better understanding of the relationship between KSHV and host cells, specifically, how KSHV infection affects the production of host miRNA and their downstream targets, as well as its underlying mechanisms, will enable us to develop novel interventions against KSHV infection.

Our previously microarray study showed that the expression of MAP4K4 was upregulated in KS biopsies[21]; however, downregulated certain let-7 family miRNAs were downregulated family [19] in KS biopsies were noticed. In silico analysis designated MAP4K4 as potential target of some let-7 miRNAs family. It is therefore of significance to study the interaction between let-7 miRNAs family and kinase MAP4K4. In our current study, we demonstrate that miRNA let-7a can inhibit KSHV reactivation by regulating the expression of MAP4K4 and the activation of MAPK signaling pathways.

## Materials and Methods

### Patients and tissue specimens

The diagnosis of KS without acquired immunodeficiency syndrome (AIDS) was based on clinical, pathologic, serologic and histological criteria. Clinical data for KS patients were obtained by medical record review approved by the Human Study Ethic Committees at the Affiliated Hospital of Hangzhou Normal University. The collection of samples was approved by the Institutional Review Board of Hangzhou Normal University and the Human Study Ethic Committees at the Affiliated Hospital of Hangzhou Normal University. Skin biopsy specimens from healthy spots and lesion spots from 4 KS patients were collected after written informed consent was obtained from participants. Biopsy specimens were snap frozen in Optimal Cutting Temperature compound immediately after resection and stored at −80°C to extract RNA for real-time PCR.

### Cell culture and KSHV preparation

Body-cavity-based lymphoma cell line 1 (BCBL-1) cells were cultured in RPMI 1640 medium supplemented with 10% (v/v) fetal calf serum (FCS), 2mM glutamine, 1 mM sodium pyruvate, 100 U/ml of penicillin and 100 mg/ml of streptomycin at 37^°^C under 5% CO_2_. 293T cells were cultured in DMEM medium supplemented with 10% (v/v) of FCS, 2mM glutamine, 1 mM sodium pyruvate, 100 U/ml penicillin and 100 mg/ml streptomycin at 37^°^C under 5% CO_2_. BC-3 cells were cultured in RPMI 1640 medium supplemented with 20% (v/v) of FCS, other condition is the same as 293T cells.

For KSHV generation, BCBL-1 cells were grown to a density of 3×10^5^ cells/ml, 12-O-tetradecanoyl-phorbol-13-acetate (TPA) was added to a final concentration of 20ng/ml for 72h and the cells and supernatant were collected separately by brief centrifugation. Collected cells were further lysed through freezing and thawing by adding liquid nitrogen 3 times. The pellet was re-suspended with the supernatant and filtered through a 0.22um filter to remove the debris and collect the flow through. This was centrifuged at 150000×g, 4°C, for 2hrs, the supernatant was removed and the pellet air dried. The viruses were re-suspended in DMEM medium without antibiotics or FBS.

### Plasmids

The 248 bp MAP4K4 3′ UTR fragment containing the let-7 binding site was amplified by PCR with specific primers (Table 1, UTRFor and UTRRev) and cloned into the pmirGLO dual-luciferase miRNA target expression vector (Promega, Madison, WI, USA) to get the wt-pmirGLO-MAP4K4-3’utr plasmid. The point mutations were generated by PCR using pmirGLO-MAP4K4-wt-3’utr as a template based on instructions (QuikChange II XL Site-Directed Mutagenesis, Stratagene, La Jolla, CA, USA), with specific mutagenic primers (Table 1, UTRmutFor and UTRmutRev) and confirmed by nucleotide sequencing. The target site for microRNA let-7a (5’-CTA**cc**TCA-3’) was mutated to 5’-CTA**gg**TCA-3, to create mt-pmirGLO-MAP4K4- 3’utr. The let-7a encoding fragment was amplified by PCR using DNA extracted from 293T cells with specific primers (Table 1, LetFor and LetRev) and cloned into pSilencer 4.1 vector (Invitrogen, Carlsbad, CA, USA). The let-7 sponge fragment, containing the complementary sequence of let-7, was cloned into the pEGFP-C2 vector, to create pEGFP-C2-let-7 sponge. The MAP4K4 ORF was amplified with specific primers (Table 1, ORFFor and ORFRev) and cloned into the pIRES2-EGFP vector to create the pIRES2-EGFP-MAP4K4 construct. The KSHV ORF50 was amplified from BCBL-1 DNA with specific primers (Table 1, ORF50For and ORF50Rev) and cloned into pIRES2-EGFP vector. In this study, the pIRES2-EGFP-ORF50 was used as the standard to calculate KSHV viral load quantity.

**Table 1:** Primers used for this project.

name	sequence
UTRwtFor	5’gcggagctcAGAACTTTGTCGTGTG3’SacI
UTRwtRev	5’ccgctctagaATCTGGTCGTTTTGGT3’XbaI
UTRmutFor	5’CCTATTTTTTTTTTccTAGGTCATTGTTCTTAATG3’
UTRmutRev	5’GGATTAAGAA CAATGAGGTAggCAAAAAAAAAAT3’
LetFor	5’cccggatccCCTGGATGTTCTCTTCACTG3’BamHI
ORFRev	5’ cggaattcCTACCAGCTCAGAAGAGAAGTCCTGCCT 3’EcoRI
ORF50For	5’ CATctcgagATGAAAGAATGTTCCAA 3’XhoⅠ
ORF50Rev	5’ CATggatccTCAGTCTCGGAAGTAATTACG 3’BamHⅠ
MAP4K4For	5’ TTGCCTACCTCATTGTTCTT 3’
MAP4K4Rev	5’ TTCTTGTGCCTGCTGATT 3’
GAPDHFor	5’ GTCGGAGTCAACGGATTTGG 3’
GAPDHRev	5’ AAGCTTCCCGTTCTCAGCCT 3’

### Viral load assay

To determine the intracellular viral load, cells were harvested at the indicated time points and were further lysed through freezing and thawing by adding liquid nitrogen 3 times. The pellet was re-suspended with the supernatant and filtered through a 0.22um filter to remove the debris and collect the flow through then treated with DNase I (Invitrogen, Carlsbad, CA, USA) at a concentration of 100 U/ml for 1 h. After heat inactivation of DNase I at 65°C for 30 min in the presence of 10 mM EDTA, ORF50 primers (5’- CGCAATGCGTTACGTTGTTG-3’ and 5’-GCCCGGACTGTTGAATCG-3’) were used to determine the presence of viral genomes, and genome copies were quantified by comparison with an ORF50 plasmid-derived standard curve.

### KSHV infection assay

The 293T cells were seeded in 12-well plates (80,000 cells/well) and cultured with complete DMEM media. 24hrs later, the supernatant was removed and the cells were washed with PBS once, and 100ul KSHV (about 5ug DNA), mixed with 900 μl DMEM without FBS, were added to each well. The plate was centrifuged at 2,500 rpm for 30min at room temperature, and returned to the incubator. The KSHV media was removed 4hrs later, and the cells washed with PBS once. The cells were cultured with fresh DMEM, and collected 24, 48, 72, 96, 120, 144hr later. The mRNA levels of KSHV, RTA, and LANA were detected by RT-PCR.

### Luciferase activity assay

Doses of pSilencer 4.1-Let-7a and pmirGLO-MAP4K4-wt-3’utr or pmirGLO-MAP4K4-mt-3’utr were co-transfected into 293T cells using lipofactamine 2000 reagent (Invitrogen, Carlsbad, CA, USA). Cells were cultured in DMEM for 24 hrs, then collected and analyzed for luciferase activity. Luciferase activity was measured using the dual-luciferase reporter assay kit (Promega, Madison, WI, USA) and normalized to *renilla* luciferase activity and total protein level.

### Real-time PCR

To measure the production of let-7 miRNA, cells were harvested and miRNAs were isolated using miRcute miRNA Isolation Kit (Tiangen Biotech, Beijing, China). The let-7 miRNAs were quantified using the methods as described previously (Takara Bio Inc. Otsu, Japan) with commercial primers (Ruibo, Guangzhou, China). Data were analyzed by delta–delta *C*_t_ (ΔΔ*C*_t_) method [22].

Total RNA from cells was reverse transcribed using the Thermoscript RT-PCR System (Invitrogen, Carlsbad, CA, USA). The MAP4K4 mRNA was assayed by real-time PCR performed by the methods described previously (Takara Bio Inc. Otsu, Japan) with specific primers (Table 1, MAP4K4For, MAP4K4Rev). Real-time PCR data were presented using ΔΔ*C*_t_ method with GAPDH (Table 1, primers GAPDHFor and GAPDHRev) as the internal control.

### Western blots

All western blots were performed with specific antibodies: MAP4K4, phospho-MAP4K4, ERK, phospho-ERK, JNK, phosphor-JNK, and p38 and phosphor-p38 (Abcam, Cambridge, MA, USA), beta-actin (Santa Cruz Biotechnology, Santa Cruz, CA, USA) was used as the internal control.

## Results

### The expression of miRNA Let-7a is increased along with decreased expression of MAP4K4 in KS patients

**Fig 1:**
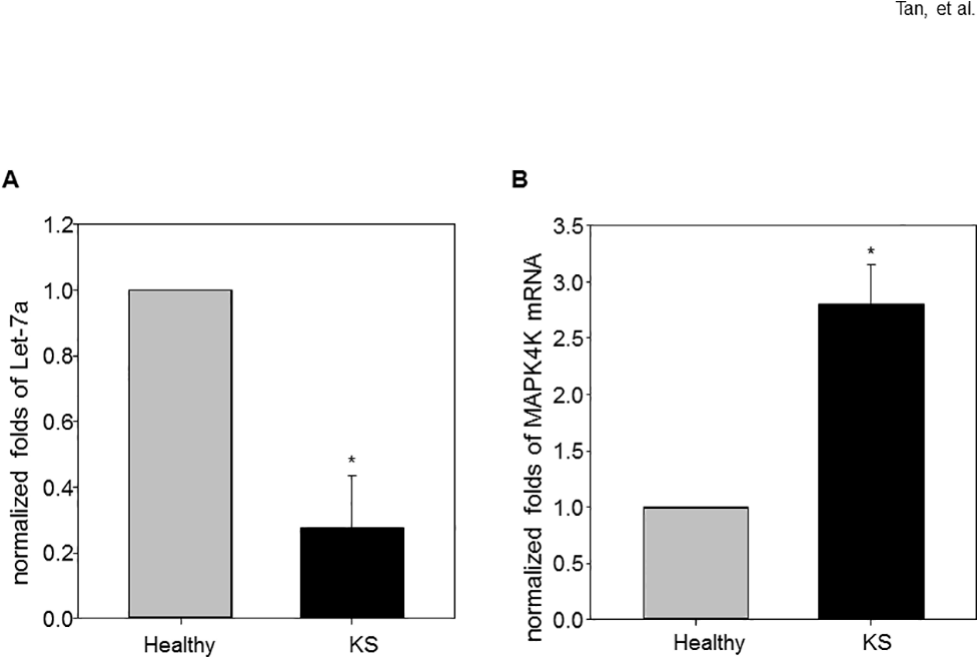
Decreased let-7 miRNA accompanies enhanced MAP4K4 expression in KS lesions. (A) The expression of let-7a miRNA was significantly decreased in KS lesioned skin compared to normal skin; (B) expression of MAP4K4 was increased in KS lesioned skin compared to normal skin. Data are expressed as means ± SEM (N = 4). Statistical differences of experimental group versus control group are reported. Data are pooled from three independent experiments. *p < 0.05.

We examined the expression of microRNA let-7 family and MAP4K4 in biopsy samples from healthy and KS patients. Real-time PCR analyses showed that lesioned skin from the same KS patients contained significantly decreased Let-7a, and increased MAP4K4 expression at the mRNA level compared with tissue from normal subjects (Fig 1A and 1B). All the KS tumors are KSHV LANA positive by Immunohistochemistry assay (data not shown). Expression of other let-7 family members are also decreased significantly (S1 Fig).

### Decreased let-7 miRNA accompanied enhanced MAP4K4 expression in KSHV infected 293T cells

**Fig 2:**
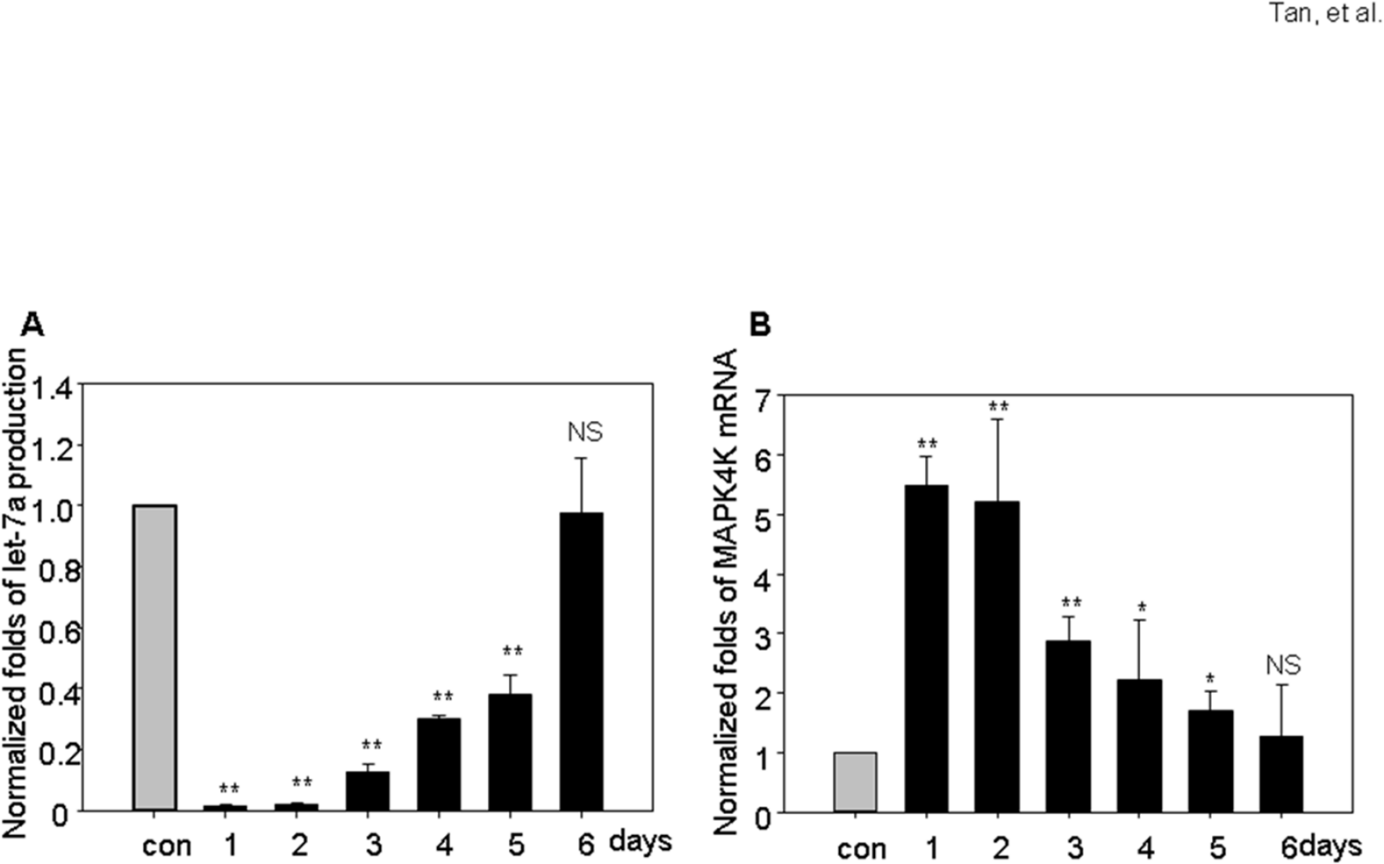
Decreased let-7 miRNA accompanies enhanced MAP4K4 expression in KSHV infected 293T cells. The expression of let-7a miRNA was significantly inhibited during 1–3 days post KSHV infection, and restored to normal level in day 6 (A). MAP4K4 mRNA production increased during the first 4 days post infection of KSHV (B). Data are expressed as means ± SEM. Statistical differences of experimental group versus control group are reported. Data are pooled from three independent experiments. *p < 0.05, **p < 0.01.

In 293T cells infected with generated KSHV, the let-7a miRNA was dramatically decreased up to 5 days post infection, but restored to normal on day 6 post-infection (Fig 2A). Similar expression patterns were noticed for other let-7 miRNA members (S2 Fig). The MAP4K4 mRNA level is increased dramatically after KSHV infection then gradually returned to normal levels at day 5 and day 6 (Fig 2B).

### Let-7a suppresses MAP4K4 expression

**Fig 3:**
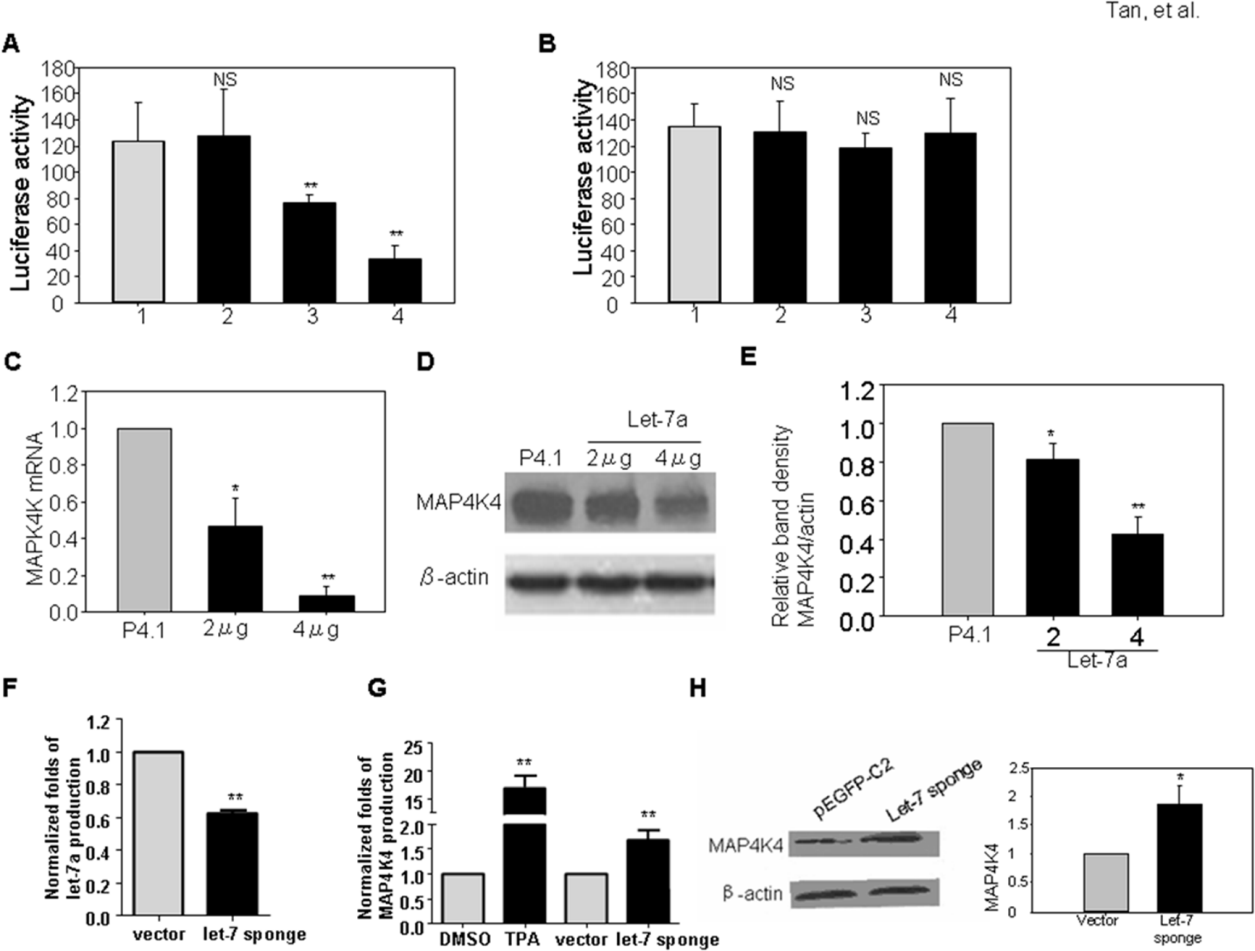
Let-7a targets MAP4K4 3’UTR and suppresses MAP4K4 expression at both mRNA and protein level. Doses of let-7a or empty psilencer4.1 and wt-MAP4K4 3’UTR or mt-MAP4K4 3’UTR were cotransfected into 293T cells, and the luciferase activity was measured. (A) Let-7a suppresses wt-MAP4K4 3’UTR activity in a dose dependent manner; however, (B) Let-7a has no suppression on mt-MAP4K4 3’UTR activity. Over expression of let-7a decreases the expression of MAP4K4 at mRNA (C) and protein (D and E) levels in 293T. Let-7 sponge or empty vector pEGFP-C2 were transfected into 293T cells and the production of let-7a and MAP4K4 were detected. (F) let-7 sponge decreases let-7a production, but does not alter the expression of MAP4K4 mRNA (G) and protein (H) levels in BCBL-1 cells. Data are representative of three independent experiments. Error bars represent the means ± SEM. The *p* values were determined by Student *t* test. *p < 0.05, **p < 0.01.

The predicted binding sites for let-7 family miRNAs within the 3’-UTR of MAP4K4 were analyzed by DIANA-microT v3.0 (DIANA—microT v3.0)[23–24]. All let-7 miRNAs (let-7a, b, c, d, e, f, g, i and mir-98) have similar mature sequences and can complementarily bind to the 3’-UTR region of MAP4K4 (S3 Fig). Let-7a is down regulated in KS tumor tissue and also reduced most sharply during the first 2 days with KSHV infection in 293T cells (Figs 1A and 2B). In this study, we picked let-7a for further investigation. To study the association of let-7a with its potential binding site in MAP4K4 3’-UTR, luciferease assays (Fig 3A) were performed. We found that let-7a can suppress the luciferase activity of the wild-type MAP4K4 3’UTR in a dose dependent manner, but has no effect on the mutated MAP4K4 3’UTR (Fig 3B). To further investigate whether let-7a can block MAP4K4 expression, the MAP4K4 mRNA and protein were analyzed in let-7a over-expressed 293T cells. qRT-PCR and western-blot assays revealed that let-7a inhibits significantly the expression of MAP4K4 at both mRNA and protein levels (Fig 3C, 3D and 3E). Similar results were found in lymphoma cell line BC-3 and lymphoblast cell line BCBL-1 (S4 Fig).

To confirm the effect of let-7 on MAP4K4 expression, we constructed the let-7 sponge expression vector and transfected BCBL-1 cells. The let-7a (Fig 3F) and other miRNAs of the let-7 family (data not shown) were down-regulated; MAP4K4 mRNA levels are slightly increased, and the protein productions in these cells were elevated when silencing let-7(Fig 3G and 3H).

### MAP4K4 and let-7a regulate MAPK signaling pathways

**Fig 4:**
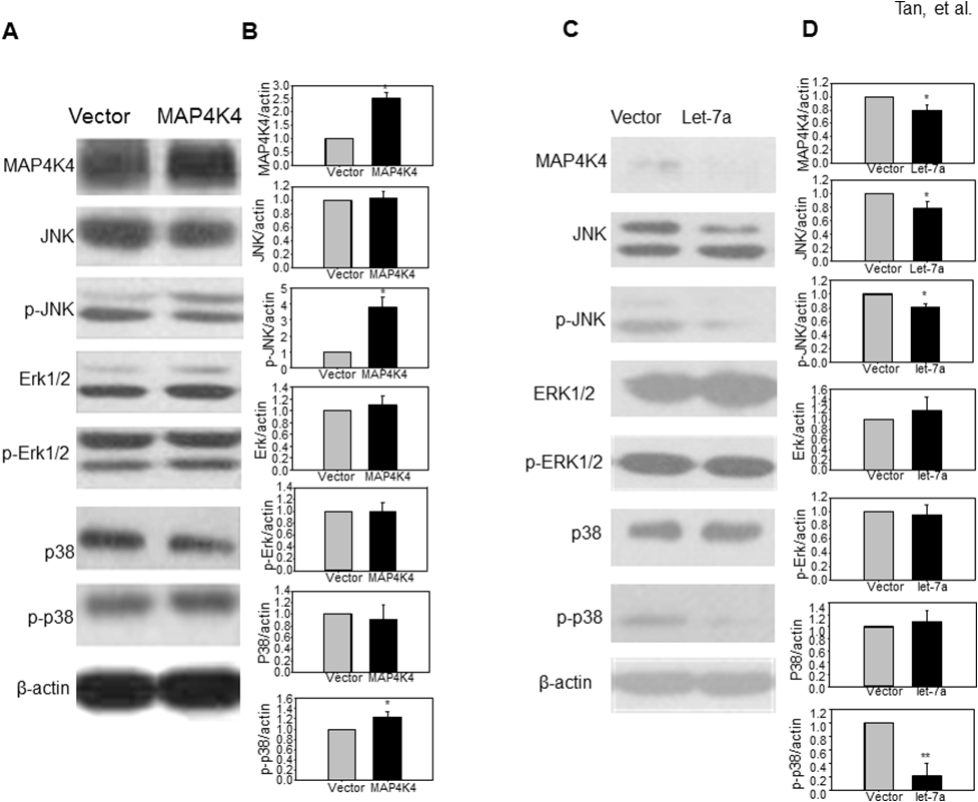
Let-7a reverses the stimulation of MAP4K4 on JNK and p38 signaling in BC-3 cells. Western blots were performed with specific antibodies to detect the phosphorylation status of JNK, ERK1/2 and p38, and the expression of total JNK, ERK1/2 and p38 in BC-3 cells transfected with pEGFP-N3-MAP4K4 (MAP4K4) or psliencer4.1-let-7a (let-7a) for 48hrs. The β-actin antibody functions as an internal control. (A and B) In BC-3 cells, overexpression of MAP4K4 can stimulate the phosphorylation of JNK and p38; however, (C and D) over expression of let-7a reduces MAP4K4 expression, JNK expression, and reduce the phosphorylation levels of JNK and p38.

MAPK pathways, such as ERK1/2, JNK, and p38 pathways, have been reported to be required for KSHV reactivation [25–27]. Given the data that let-7a can decrease the expression of MAP4K4, we investigated the effect of let-7a and MAP4K4 on ERK1/2, JNK, and p38 protein and their phosphorylation status. In BC-3 cells with overexpressed MAP4K4, the phosphorylated JNK and p38 level were increased (Fig 4A and 4B). Over- expression of let-7a reduced MAP4K4 expression, the JNK protein level, and the phosphorylated level of JNK, and p38 (Fig 4C and 4D).

### Let-7a suppresses KSHV reactivation, while MAP4K4 and let-7 sponge induces KSHV reactivation in BC-3 cells

**Fig 5:**
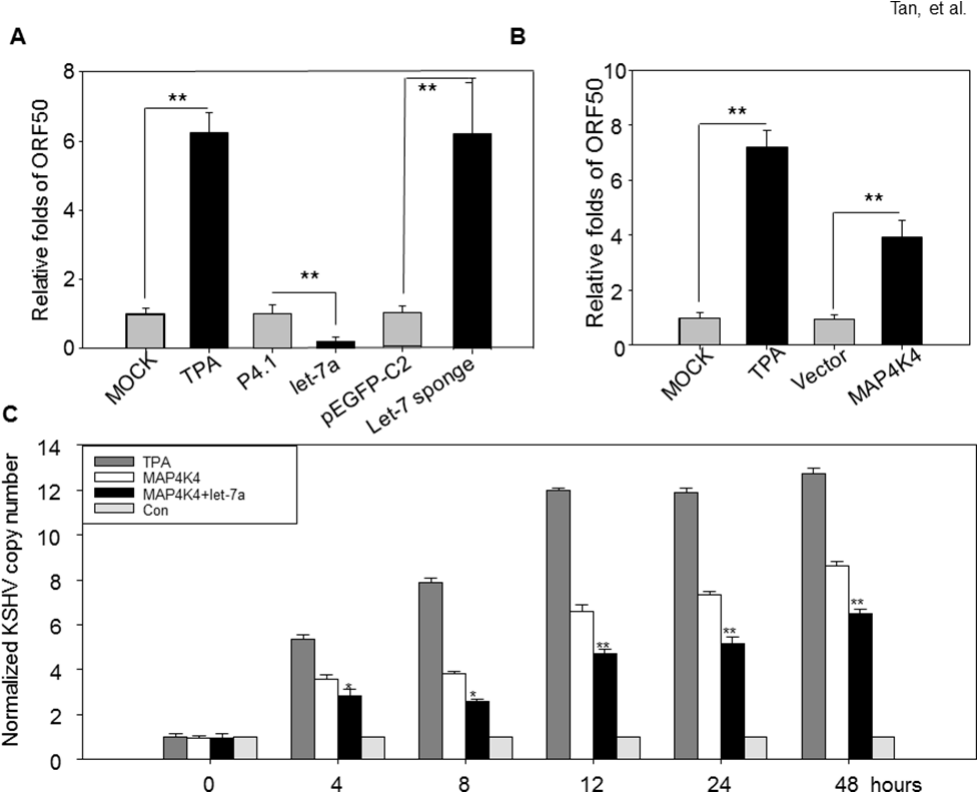
Let-7a reverses the KSHV reactivation induced by MAP4K4. (A) BC-3 cells were transiently transfected with 2.0μg of psilencer4.1-let-7a (let7a) and let-7 sponge or corresponding empty vector (psilencer4.1 or pEGFP-C2), the KSHV ORF50 copy number was quantified by qRT-PCR. (B) BC-3 cells transfected with 2.0μg pEGFP-N3- MAP4K4 or empty vector pEGFP-N3, the KSHV ORF50 copy number was quantified by qPRT-PCR. BC-3 cells treated with 20ng/ml of TPA were used as positive control. (C) BC-3 cells transfected with different combination of plasmids were harvested at indicated time points, the KSHV copy number was detected by qRT-PCR.

As shown above, Let-7a suppresses JNK protein expression and phosphorylation of JNK and p38. The ERK1/2, JNK and p38 MAPK pathways are required for KSHV reactivation. We asked whether let-7a can suppress KSHV reactivation. To determine this, we quantified the KSHV ORF50 copy number in BC-3 cells with or without transfected let-7a expression vector. Compared with the BC-3 cells transfected with control plasmids, the ORF50 copy number decreased 5 fold. Silencing let-7 miRNAs, by transfecting the pEGFP-C2-let7 sponge vector, increased the ORF copy number 6.32 fold (Fig 5A). For MAP4K4, the upstream factor of MAPK signaling pathway, which is critical for KSHV reactivation, we also quantified the ORF50 copy numbers in MAP4K4 over expressed BC-3 cells; compared with the cells transfected with empty vectors, the ORF 50 copy numbers increased 3.51 fold after 48 hours of treatment (Fig 5B).

### Let-7a reverses the KSHV reactivation induced by MAP4K4

Let-7a specifically suppresses MAP4K4 expression, and has an inverse effect on JNK and p38 MAPK pathways, which are required for KSHV reactivation and replication. We hypothesis that let-7a suppresses MAP4K4-induced KSHV reactivation. We first detected the KSHV expression in BC-3 cells which over express MAP4K4. MAP4K4 induces the expression of several lytic KSHV genes, such as ORF50, K2, K4, K6, ORF7, ORF8 and vIRF2 (S5 Fig and S1 Table). The RTA encoded by ORF50 is one of the KSHV reactivation switch factor. We further quantified the KSHV ORF50 copy numbers in BCBL-1 cells which were cotransfected with let-7a and MAP4K4. The BC-3 cells treated with 20ng/ml TPA served as the positive control and BCBL-1 cells transfected with empty vectors as negative control. The copy number of ORF50 slightly increased in the negative control during the first 8 hours, then held steady through 48 hrs. MAP4K4 overexpression significantly increases the ORF 50 copy numbers, but the cotransfected let-7a reverses the ORF50 increases in a dose-dependent manner (Fig 5C).

## Discussion

In the present study, we demonstrated miRNA let-7a plays an important role in modulating KSHV reactivation through regulating the expression of MAP4K4 and thus the alteration of ERK/JNK/p38 signaling pathway. We also describe substantial new mechanistic insights into the pathogenesis of and the potential of let-7a as a novel treatment against KSHV-related malignancy.

KSHV reactivation from latency is a complicated process regulated by a very intricate relationship between viral and cellular(host) factors in different manners. Some viral factors, such as replication and transcription activator (RTA), encoded by KSHV gene *ORF50*, are known to regulate the switch from latent to lytic infection. Specifically, RTA interacts physically with recombination signal sequence-binding protein J kappa (RBP-Jkappa) (also known as CBF-1 and CSL) in the transactivation of viral promoters [28]. The RTA–RBP-Jkappa complex appears to be essential for the RTA-mediated switch from latency to lytic replication [29]. In cell culture, reactivation of KSHV occurs following treatment with chemical compounds such as the phorbol ester TPA/PMA and the histone deacetylase inhibitor sodium butyrate. Activation of Toll-like receptors (TLR) 7 and 8 in PEL cells can also lead to KSHV reactivation [30]. Both the Pim and Ras family kinases are involved in KSHV reactivation [5, 31–32]. It has been shown that early growth response-1 (Egr-1) is an essential component for the KSHV reactivation process via its ability to mediate transcription of RTA[33]. Further, studies have shown that some host factors can inhibit KSHV reactivation, such as Tousled-like Kinases[34], interferon (IFN)-α and γ [35], interferon regulatory factor 7 [36]. In addition, some other host factors can cause KSHV reactivation,for example, Pim-1 and Pim-3 contribute to viral reactivation by phosphorylating the KSHV latency associated nuclear antigen (LANA) on serine residues 205 and 206[5, 32].

The involvement of miRNA, from both KSHV and the host cell, in the regulation of latency/replication switch, has drawn increasing attention. Although a few KSHV encoded miRNAs have been shownto repress KSHV lytic reactivation, such as miR-K1 and miR-K9[29, 37], to our knowledge, host miRNAs modulating KSHV reactivation have not been well explored [20,38]. In our study, we screened the different expressed mRNAs and miRNAs in KS biopsies versus normal adjacent tissue and found that some miRNAs belong to the let-7 family. Specifically, let-7a, d, e, and i were significantly down regulated in KS tumor tissues. KSHV infection can actually inhibit the expression of all the members of Let-7 family. Even though the fact that Let-7 miRNAs function as tumor suppressors[38–39] by suppressing oncogene ras, we did not notice significantly different expressions of *kras*, *hras* and *nras* in KS biopsies in our previous studies, implying the involvement of alternative mechanisms in the let-7 related regulation of latency-replication switch. MAPK signaling pathways have been found to be critical for KSHV reactivation [28]. For example, extracellular signal-regulated kinase (ERK), Jun N-terminal kinase (JNK) and p38 are involved in KSHV reactivation [4, 25–27, 40].Interestingly, MAP4K4, containing the let-7 targeting site in its 3’UTR, is highly expressed in KS tumors compared to adjacent normal tissues. MAP4K4 is the upstream regulator for ERK, JNK and p38 pathways. We hypothesized that let-7 can modulate the KSHV latent/lytic switch, and regulating ERK, JNK and p38 pathways by alternate MAP4K4 expression levels. The Raf/MEK/ERK/Ets-1 pathway is one of the important pathways mediating KSHV reactivation [26, 31]. In primary infection of HUVEC, KSHV activates MEK/ERK, JNK, and p38 MAPK pathways, all of which then activate AP-1, to facilitate its entry into target cells and productive lytic replication at the early acute stage of infection [27, 41]. In the present study, we confirm that MAP4K4 is directly targeted by let-7a. Over expression of let-7a suppresses KSHV replication, while MAP4K4 induces KSHV reactivation. A recent paper reported that MAP4K4 is a pivotal factor for inducing KSHV reactivation[42]. The phosphorylation levels of JNK and p38 were slightly altered in BCBL-1 cells over-expressing MAP4K4, while JNK and ERK phosphorylation levels were also slightly changed in let-7a over-expressing BCBL-1 cells. Interestingly, let-7 sponge, silencing let-7a and other let-7 family miRNAs, did not increase MAP4K4 expression. These results suggest that, besides MAP4K4, other factors may also be involved in modulating KSHV reactivation by let-7.

In conclusion, this study showed that the reactivation of KSHV by MAP4K4 was inhibited by let-7a, implying that let-7a modulates the KSHV latency/lytic switch by regulating MAP4K4 expression and its related MAPK pathways. Other factors may also play a role in regulating KSHV reactivation by let-7.

## Supporting Information

S1 Fig KS lesion has increased expression of miRNA let-7 family, such as let-7d, 7e and 7i. The expression of miRNAs let-7d (A), let-7e (B) and let-7i (C) were significantly decreased in KS lesioned skin compared to normal skin. Data are expressed as means ± SEM (N = 4). Statistical differences of experimental group versus control group are reported. Data are pooled from three independent experiments. *p < 0.05.
(TIF)

S2 Fig The expression of other miRNA let-7 family members demonstrated similar patterns in comparison with let-7a. The expression of miRNAs let-7b (A), let-7c (B), let-7d (C), let-7e (D), let-7f (E), let-7g (F), let-7i (G) and mir-98 (H) were significantly inhibited during the indicated days after KSHV infection, which could be restored at different levels and different days. Data are expressed as means ± SEM. Statistical differences of experimental group versus control group are reported. Data are pooled from three independent experiments. *p < 0.05, **p < 0.01, NS represents no significance.
(TIF)

S3 Fig All let-7 miRNAs (let-7a, b, c, d, e, f, g, i and mir-98) have similar mature sequences and can complementary bind to 3’-UTR region of MAP4K4. 3’-UTR sequence of MAP4K4 and the mature miRNAs sequences of let-7a, b, c, d, e, f, g, i and mir-98 were shown and the complementally sequences were highlighted.
(TIF)

S4 Fig let-7a inhibits MAP4K4 in BC-3 and BCBL-1 cells. qRT-PCR demonstrated that let-7a inhibits significantly the transcripts of MAP4K4 in lymphoma cell line BC-3 (A)and lymphoblast cell line BCBL-1(B). TPA here functions as control, which can stimulate MAP4K4 expression instead. *p < 0.05.
(TIF)

S5 Fig MAP4K4 induces the expression of lytic and latency KSHV genes. Total RNA was extracted from MAP4K4-transfected BCBL-1 cells, and then KSHV latency (ORF50, K2, K6, ORF 7, ORF 8 and vIRF2) and lytic (ORF72, ORF73 and K13) related genes were detected by reverse transcript PCR and visualized by DNA electrophoresis. Total RNA obtained from DMSO treated BCBL-1 cells functions as negative controls, and total RNAs from TPA treated BCBL-1 was as positive controls. The primers used in these PCRs were listed in S1 Table.
(TIF)

S1 Table Primer sequences used for RT-PCR for KSHV genes. (TIF)

## References

[1] Chang Y, Cesarman E, Pessin MS, Lee F, Culpepper J, Knowles DM, et al Identification of herpesvirus-like DNA sequences in AIDS-associated Kaposi's sarcoma. Science. 1994;266(5192):1865–9. Epub 1994/12/16. . 7997879 10.1126/science.7997879

[2] Cesarman E, Chang Y, Moore PS, Said JW, Knowles DM. Kaposi's sarcoma-associated herpesvirus-like DNA sequences in AIDS-related body-cavity-based lymphomas. N Engl J Med. 1995;332(18):1186–91. Epub 1995/05/04. . 7700311 10.1056/NEJM199505043321802

[3] Greene W, Kuhne K, Ye F, Chen J, Zhou F, Lei X, et al Molecular biology of KSHV in relation to AIDS-associated oncogenesis. Cancer treatment and research. 2007;133:69–127. 17672038 10.1007/978-0-387-46816-7_3 PMC2798888

[4] Xie J, Ajibade AO, Ye F, Kuhne K, Gao SJ. Reactivation of Kaposi's sarcoma-associated herpesvirus from latency requires MEK/ERK, JNK and p38 multiple mitogen-activated protein kinase pathways. Virology. 2008;371(1):139–54. Epub 2007/10/30. doi: S0042-6822(07)00635-6 [pii] 10.1016/j.virol.2007.09.040 17964626 PMC2239004

[5] Cheng F, Weidner-Glunde M, Varjosalo M, Rainio EM, Lehtonen A, Schulz TF, et al KSHV reactivation from latency requires Pim-1 and Pim-3 kinases to inactivate the latency-associated nuclear antigen LANA. PLoS Pathog. 2009;5(3):e1000324 Epub 2009/03/07. 10.1371/journal.ppat.1000324 19266083 PMC2648312

[6] Grundhoff A, Ganem D. Inefficient establishment of KSHV latency suggests an additional role for continued lytic replication in Kaposi sarcoma pathogenesis. The Journal of clinical investigation. 2004;113(1):124–36. 10.1172/JCI17803 14702116 PMC300762

[7] Wang HW, Trotter MW, Lagos D, Bourboulia D, Henderson S, Makinen T, et al Kaposi sarcoma herpesvirus-induced cellular reprogramming contributes to the lymphatic endothelial gene expression in Kaposi sarcoma. Nat Genet. 2004;36(7):687–93. Epub 2004/06/29. doi: 10.1038/ng1384 ng1384 [pii]. . 15220918 10.1038/ng1384

[8] Ye FC, Blackbourn DJ, Mengel M, Xie JP, Qian LW, Greene W, et al Kaposi's sarcoma-associated herpesvirus promotes angiogenesis by inducing angiopoietin-2 expression via AP-1 and Ets1. J Virol. 2007;81(8):3980–91. Epub 2007/02/09. doi: JVI.02089-06 [pii] 10.1128/JVI.02089-06 17287278 PMC1866109

[9] Ambros V. microRNAs: tiny regulators with great potential. Cell. 2001;107(7):823–6. . 11779458 10.1016/s0092-8674(01)00616-x

[10] Bartel DP. MicroRNAs: genomics, biogenesis, mechanism, and function. Cell. 2004;116(2):281–97. Epub 2004/01/28. doi: S0092867404000455 [pii]. . 14744438 10.1016/s0092-8674(04)00045-5

[11] Ambros V. The functions of animal microRNAs. Nature. 2004;431(7006):350–5. Epub 2004/09/17. 10.1038/nature02871 nature02871 [pii]. 15372042. 15372042

[12] Filipowicz W, Bhattacharyya SN, Sonenberg N. Mechanisms of post-transcriptional regulation by microRNAs: are the answers in sight? Nat Rev Genet. 2008;9(2):102–14. Epub 2008/01/17. doi: nrg2290 [pii] 10.1038/nrg2290 . 18197166

[13] Lewis BP, Shih IH, Jones-Rhoades MW, Bartel DP, Burge CB. Prediction of mammalian microRNA targets. Cell. 2003;115(7):787–98. Epub 2003/12/31. doi: S0092867403010183 [pii]. . 14697198 10.1016/s0092-8674(03)01018-3

[14] Fu M, Gao Y, Zhou Q, Zhang Q, Peng Y, Tian K, et al Human cytomegalovirus latent infection alters the expression of cellular and viral microRNA. Gene. 2014;536(2):272–8. Epub 2013/12/24. doi: S0378-1119(13)01673-9 [pii] 10.1016/j.gene.2013.12.012 . 24361963

[15] Zhao G, Zhang JG, Liu Y, Qin Q, Wang B, Tian K, et al miR-148b functions as a tumor suppressor in pancreatic cancer by targeting AMPKalpha1. Mol Cancer Ther. 2013;12(1):83–93. Epub 2012/11/23. doi: 1535-7163.MCT-12-0534-T [pii] 10.1158/1535-7163.MCT-12-0534-T . 23171948

[16] Liang D, Lin X, Lan K. Looking at Kaposi's Sarcoma-Associated Herpesvirus-Host Interactions from a microRNA Viewpoint. Front Microbiol. 2011;2:271 Epub 2012/01/26. 10.3389/fmicb.2011.00271 22275910 PMC3258008

[17] Catrina Ene AM, Borze I, Guled M, Costache M, Leen G, Sajin M, et al MicroRNA Expression Profiles in Kaposi's Sarcoma. Pathol Oncol Res. 2014;20(1):153–9. Epub 2013/09/13. 10.1007/s12253-013-9678-1 . 24027049

[18] Chugh PE, Sin SH, Ozgur S, Henry DH, Menezes P, Griffith J, et al Systemically Circulating Viral and Tumor-Derived MicroRNAs in KSHV-Associated Malignancies. PLoS Pathog. 2013;9(7):e1003484 Epub 2013/07/23. 10.1371/journal.ppat.1003484 PPATHOGENS-D-12-02933 [pii]. 23874201 PMC3715412

[19] O'Hara AJ, Wang L, Dezube BJ, Harrington WJ Jr., Damania B, Dittmer DP. Tumor suppressor microRNAs are underrepresented in primary effusion lymphoma and Kaposi sarcoma. Blood. 2009;113(23):5938–41. 10.1182/blood-2008-09-179168 19252139 PMC2700328

[20] Yan Q, Li W, Tang Q, Yao S, Lv Z, Feng N, et al Cellular microRNAs 498 and 320d regulate herpes simplex virus 1 induction of Kaposi's sarcoma-associated herpesvirus lytic replication by targeting RTA. PLoS One. 2013;8(2):e55832 Epub 2013/02/19. doi: 10.1371/journal.pone.0055832 PONE-D-12-31366 [pii]. 23418466 10.1371/journal.pone.0055832 PMC3572171

[21] Tan X, Li D, Wang X, Zeng Y, Yan Y, Yang L. Claudin-2 downregulation by KSHV infection is involved in the regulation of endothelial barrier function. J Cutan Pathol. 2014;41(8):630–9. Epub 2014/07/06. 10.1111/cup.12332 . 24995964

[22] Dittmer DP. Transcription profile of Kaposi's sarcoma-associated herpesvirus in primary Kaposi's sarcoma lesions as determined by real-time PCR arrays. Cancer research. 2003;63(9):2010–5. . 12727810

[23] Maragkakis M, Alexiou P, Papadopoulos GL, Reczko M, Dalamagas T, Giannopoulos G, et al Accurate microRNA target prediction correlates with protein repression levels. BMC Bioinformatics. 2009;10:295. Epub 2009/09/22. doi: 1471-2105-10-295 [pii] 10.1186/1471-2105-10-295 19765283 PMC2752464

[24] Maragkakis M, Reczko M, Simossis VA, Alexiou P, Papadopoulos GL, Dalamagas T, et al DIANA-microT web server: elucidating microRNA functions through target prediction. Nucleic Acids Res. 2009;37(Web Server issue):W273–6. Epub 2009/05/02. doi: gkp292 [pii] 10.1093/nar/gkp292 19406924 PMC2703977

[25] Sharma-Walia N, Krishnan HH, Naranatt PP, Zeng L, Smith MS, Chandran B. ERK1/2 and MEK1/2 induced by Kaposi's sarcoma-associated herpesvirus (human herpesvirus 8) early during infection of target cells are essential for expression of viral genes and for establishment of infection. J Virol. 2005;79(16):10308–29. Epub 2005/07/30. doi: 79/16/10308 [pii] 10.1128/JVI.79.16.10308-10329.2005 16051824 PMC1182676

[26] Ford PW, Bryan BA, Dyson OF, Weidner DA, Chintalgattu V, Akula SM. Raf/MEK/ERK signalling triggers reactivation of Kaposi's sarcoma-associated herpesvirus latency. J Gen Virol. 2006;87(Pt 5):1139–44. Epub 2006/04/11. doi: 87/5/1139 [pii] 10.1099/vir.0.81628-0 . 16603514

[27] Pan H, Xie J, Ye F, Gao SJ. Modulation of Kaposi's sarcoma-associated herpesvirus infection and replication by MEK/ERK, JNK, and p38 multiple mitogen-activated protein kinase pathways during primary infection. J Virol. 2006;80(11):5371–82. Epub 2006/05/16. doi: 80/11/5371 [pii] 10.1128/JVI.02299-05 16699017 PMC1472170

[28] Cohen A, Brodie C, Sarid R. An essential role of ERK signalling in TPA-induced reactivation of Kaposi's sarcoma-associated herpesvirus. The Journal of general virology. 2006;87(Pt 4):795–802. 10.1099/vir.0.81619-0 . 16528027

[29] Lu CC, Li Z, Chu CY, Feng J, Sun R, Rana TM. MicroRNAs encoded by Kaposi's sarcoma-associated herpesvirus regulate viral life cycle. EMBO Rep. 2010;11(10):784–90. Epub 2010/09/18. doi: embor2010132 [pii] 10.1038/embor.2010.132 20847741 PMC2948186

[30] Gregory SM, West JA, Dillon PJ, Hilscher C, Dittmer DP, Damania B. Toll-like receptor signaling controls reactivation of KSHV from latency. Proc Natl Acad Sci U S A. 2009;106(28):11725–30. Epub 2009/07/01. doi: 0905316106 [pii] 10.1073/pnas.0905316106 19564611 PMC2710638

[31] Yu F, Harada JN, Brown HJ, Deng H, Song MJ, Wu TT, et al Systematic identification of cellular signals reactivating Kaposi sarcoma-associated herpesvirus. PLoS Pathog. 2007;3(3):e44. Epub 2007/04/03. doi: 06-PLPA-RA-0231R3 [pii] 10.1371/journal.ppat.0030044 17397260 PMC1839163

[32] Bajaj BG, Verma SC, Lan K, Cotter MA, Woodman ZL, Robertson ES. KSHV encoded LANA upregulates Pim-1 and is a substrate for its kinase activity. Virology. 2006;351(1):18–28. Epub 2006/05/02. doi: S0042-6822(06)00209-1 [pii] 10.1016/j.virol.2006.03.037 . 16647097

[33] Hasan RN, Schafer AI. Hemin upregulates Egr-1 expression in vascular smooth muscle cells via reactive oxygen species ERK-1/2-Elk-1 and NF-kappaB. Circ Res. 2008;102(1):42–50. Epub 2007/10/31. doi: CIRCRESAHA.107.155143 [pii] 10.1161/CIRCRESAHA.107.155143 . 17967787

[34] Dillon PJ, Gregory SM, Tamburro K, Sanders MK, Johnson GL, Raab-Traub N, et al Tousled-like Kinases Modulate Reactivation of Gammaherpesviruses from Latency. Cell Host Microbe. 2013;13(2):204–14. Epub 2013/02/19. doi: S1931-3128(13)00033-4 [pii] 10.1016/j.chom.2012.12.005 . 23414760 PMC3602413

[35] Pozharskaya VP, Weakland LL, Offermann MK. Inhibition of infectious human herpesvirus 8 production by gamma interferon and alpha interferon in BCBL-1 cells. J Gen Virol. 2004;85(Pt 10):2779–87. Epub 2004/09/28. doi: 10.1099/vir.0.80214–0 85/10/2779 [pii]. . 15448338 10.1099/vir.0.80214-0

[36] Wang J, Zhang J, Zhang L, Harrington W Jr., West JT, Wood C. Modulation of human herpesvirus 8/Kaposi's sarcoma-associated herpesvirus replication and transcription activator transactivation by interferon regulatory factor 7. J Virol. 2005;79(4):2420–31. Epub 2005/02/01. doi: 79/4/2420 [pii] 10.1128/JVI.79.4.2420-2431.2005 15681443 PMC546578

[37] Bellare P, Ganem D. Regulation of KSHV lytic switch protein expression by a virus-encoded microRNA: an evolutionary adaptation that fine-tunes lytic reactivation. Cell Host Microbe. 2009;6(6):570–5. Epub 2009/12/17. doi: S1931-3128(09)00385-0 [pii] 10.1016/j.chom.2009.11.008 20006845 PMC2822622

[38] Slack F. let-7 microRNA reduces tumor growth. Cell cycle (Georgetown, Tex. 2009;8(12):1823. Epub 2009/04/21. doi: 8639 [pii]. . 19377282 10.4161/cc.8.12.8639

[39] Christensen BC, Moyer BJ, Avissar M, Ouellet LG, Plaza SL, McClean MD, et al A let-7 microRNA-binding site polymorphism in the KRAS 3' UTR is associated with reduced survival in oral cancers. Carcinogenesis. 2009;30(6):1003–7. Epub 2009/04/22. doi: bgp099 [pii] 10.1093/carcin/bgp099 19380522 PMC2691138

[40] DiMaio TA, Gutierrez KD, Lagunoff M. Latent KSHV infection of endothelial cells induces integrin beta3 to activate angiogenic phenotypes. PLoS Pathog. 2011;7(12):e1002424 Epub 2011/12/17. doi: 10.1371/journal.ppat.1002424 PPATHOGENS-D-11-01065 [pii]. 22174684 10.1371/journal.ppat.1002424 PMC3234222

[41] Xie J, Pan H, Yoo S, Gao SJ. Kaposi's sarcoma-associated herpesvirus induction of AP-1 and interleukin 6 during primary infection mediated by multiple mitogen-activated protein kinase pathways. J Virol. 2005;79(24):15027–37. Epub 2005/11/25. doi: 79/24/15027 [pii] 10.1128/JVI.79.24.15027-15037.2005 16306573 PMC1316010

[42] Haas DA, Bala K, Busche G, Weidner-Glunde M, Santag S, Kati S, et al The inflammatory kinase MAP4K4 promotes reactivation of Kaposi's sarcoma herpesvirus and enhances the invasiveness of infected endothelial cells. PLoS Pathog. 2013;9(11):e1003737 10.1371/journal.ppat.1003737 24244164 PMC3820715

