# Supplementary figures and images for: Cellular MicroRNA Let-7a Suppresses KSHV Replication through Targeting MAP4K4 Signaling Pathways
Source: PLoS One. 2015 Jul 21;10(7):e0132148. doi: 10.1371/journal.pone.0132148 (PMC4511191; doi:10.1371/journal.pone.0132148)

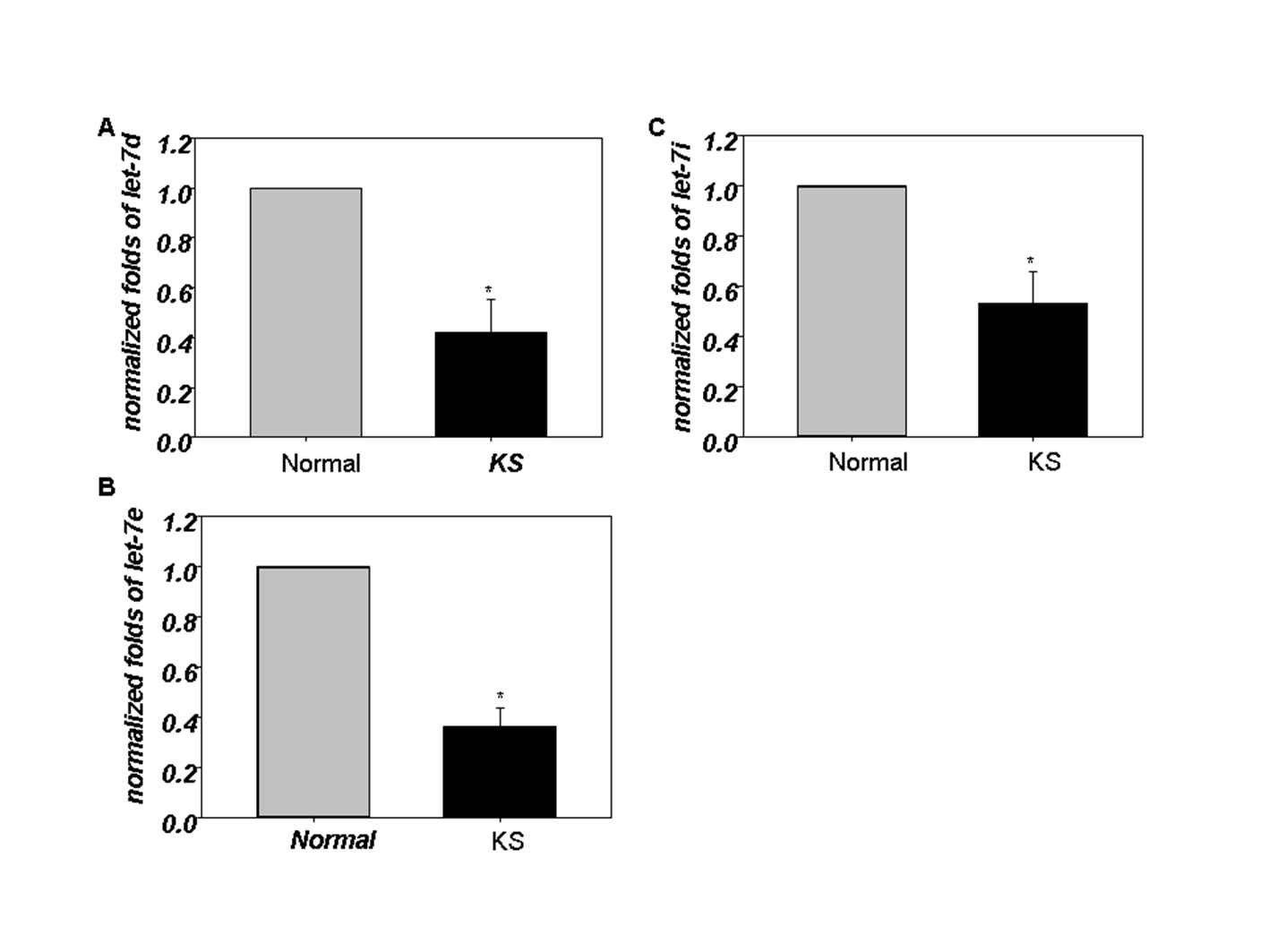

Supplement: S1 Fig — The expression of miRNAs let-7d (A), let-7e (B) and let-7i (C) were significantly decreased in KS lesioned skin compared to normal skin. Data are expressed as means ± SEM (N = 4). Statistical differences of experimental group versus control group are reported. Data are pooled from three independent experiments. *p < 0.05. (TIF) [file pone.0132148.s001.tif]

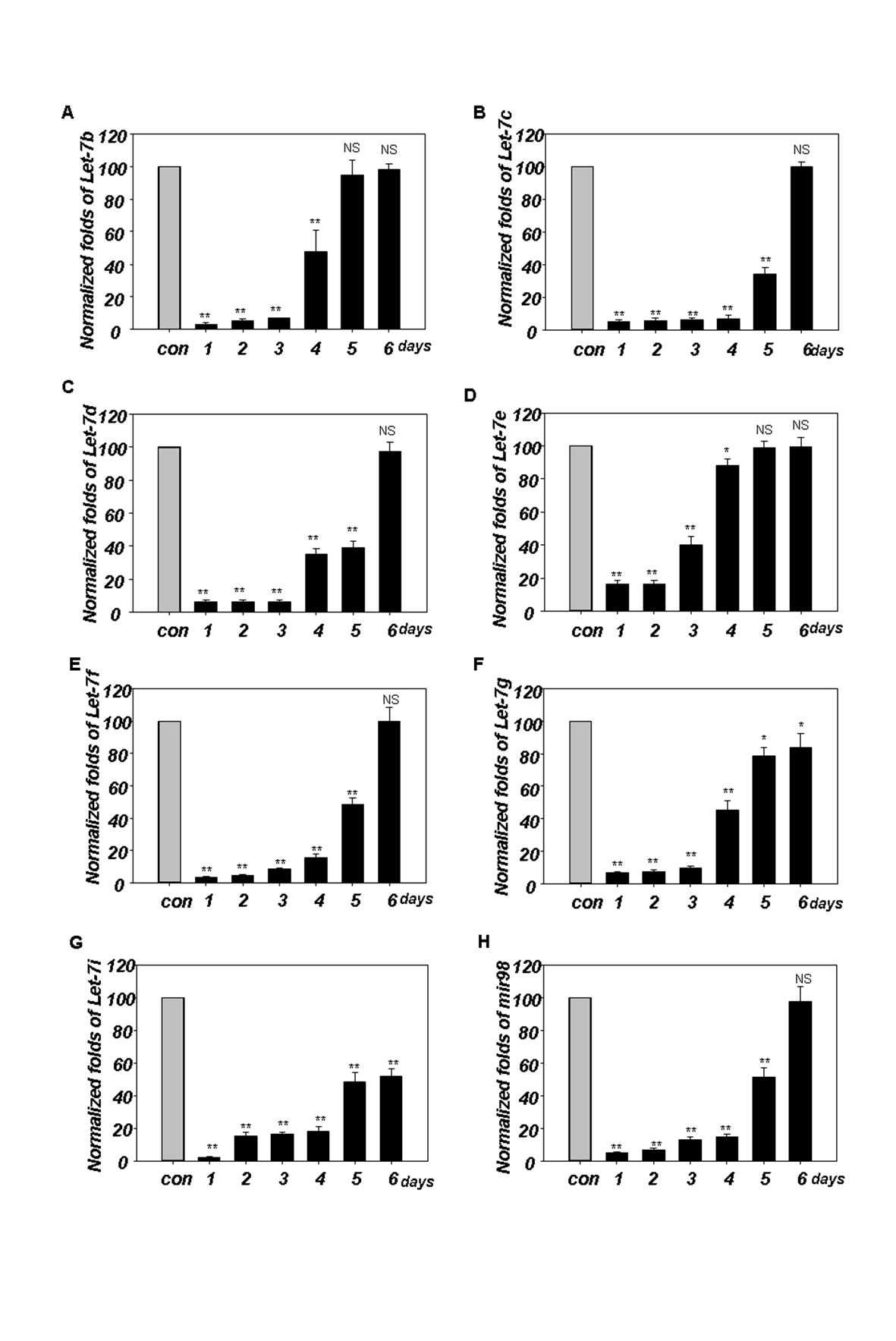

Supplement: S2 Fig — The expression of miRNAs let-7b (A), let-7c (B), let-7d (C), let-7e (D), let-7f (E), let-7g (F), let-7i (G) and mir-98 (H) were significantly inhibited during the indicated days after KSHV infection, which could be restored at different levels and different days. Data are expressed as means ± SEM. Statistical differences of experimental group versus control group are reported. Data are pooled from three independent experiments. *p < 0.05, **p < 0.01, NS represents no significance. (TIF) [file pone.0132148.s002.tif]

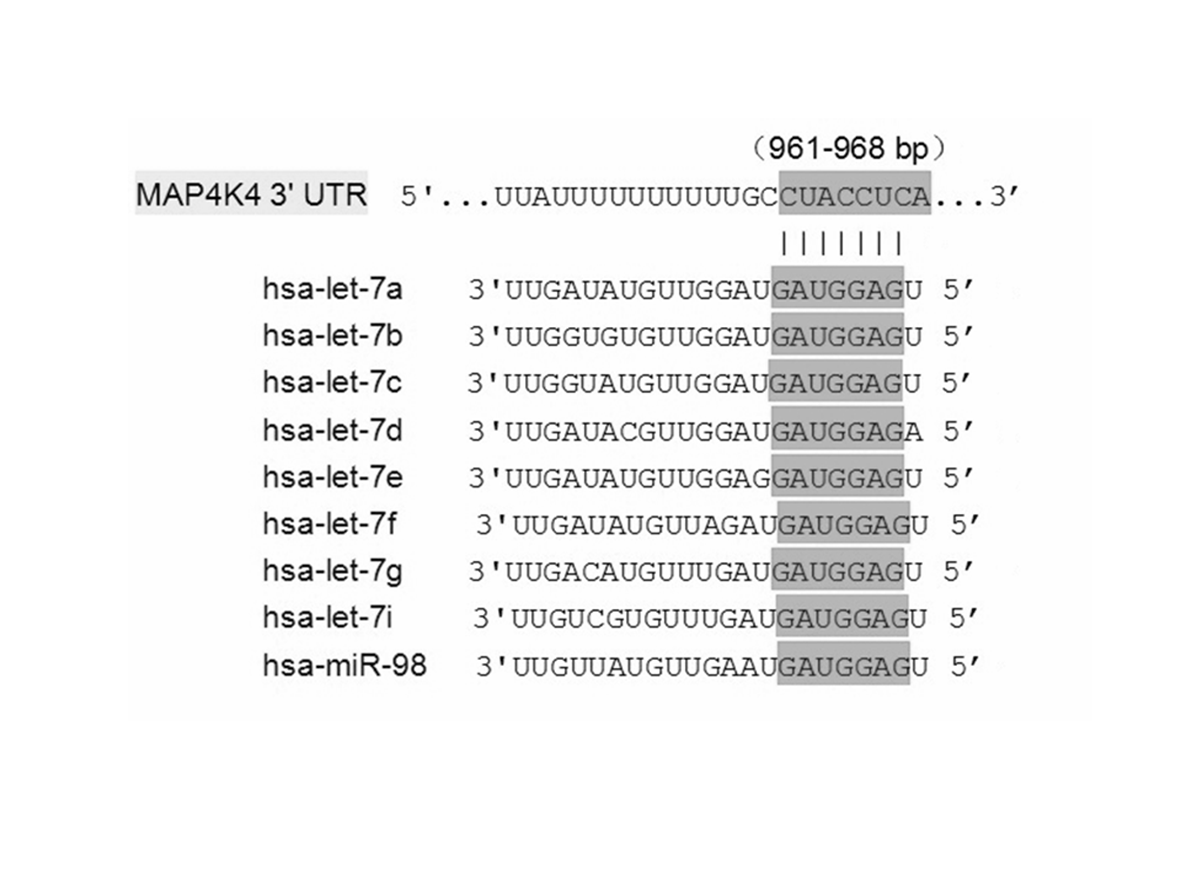

Supplement: S3 Fig — 3’-UTR sequence of MAP4K4 and the mature miRNAs sequences of let-7a, b, c, d, e, f, g, i and mir-98 were shown and the complementally sequences were highlighted. (TIF) [file pone.0132148.s003.tif]

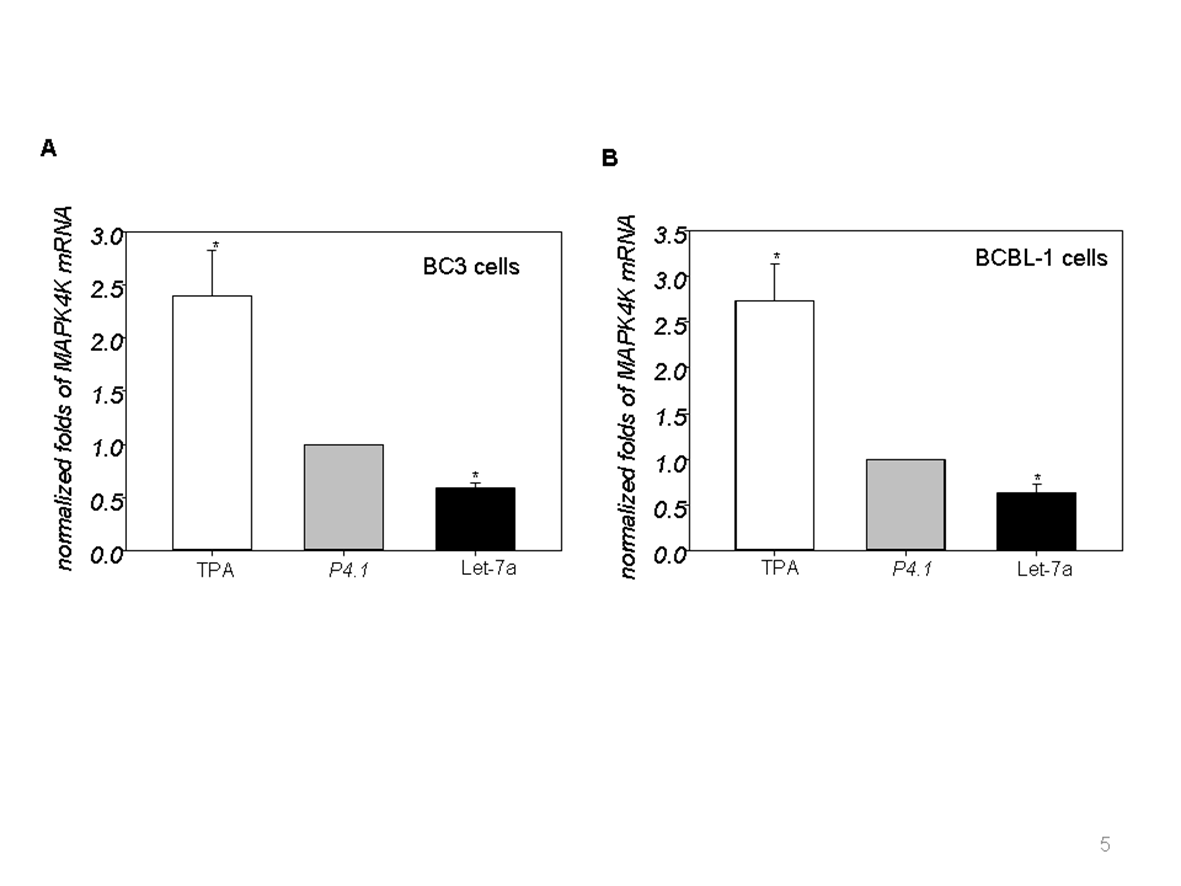

Supplement: S4 Fig — qRT-PCR demonstrated that let-7a inhibits significantly the transcripts of MAP4K4 in lymphoma cell line BC-3 (A)and lymphoblast cell line BCBL-1(B). TPA here functions as control, which can stimulate MAP4K4 expression instead. *p < 0.05. (TIF) [file pone.0132148.s004.tif]

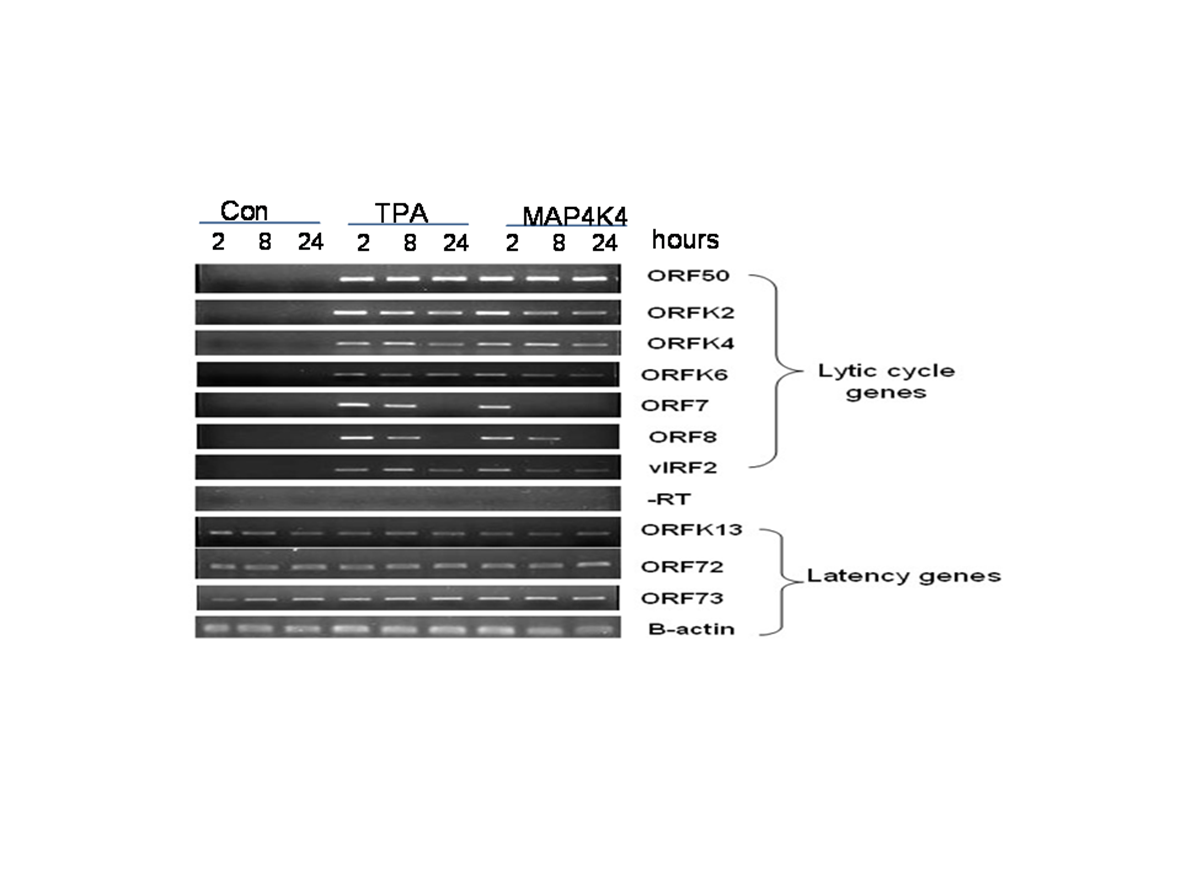

Supplement: S5 Fig — Total RNA was extracted from MAP4K4-transfected BCBL-1 cells, and then KSHV latency (ORF50, K2, K6, ORF 7, ORF 8 and vIRF2) and lytic (ORF72, ORF73 and K13) related genes were detected by reverse transcript PCR and visualized by DNA electrophoresis. Total RNA obtained from DMSO treated BCBL-1 cells functions as negative controls, and total RNAs from TPA treated BCBL-1 was as positive controls. The primers used in these PCRs were listed in S1 Table. (TIF) [file pone.0132148.s005.tif]

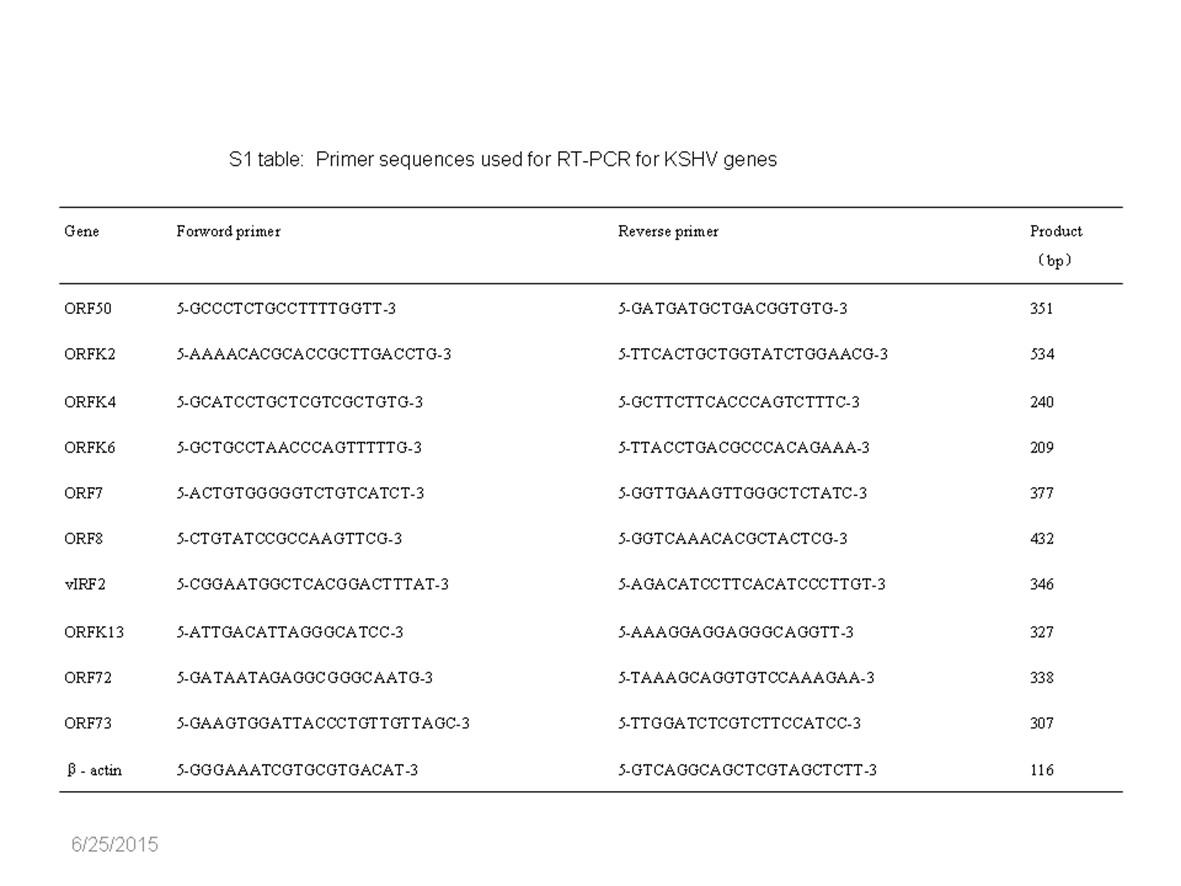

Supplement: S1 Table — (TIF) [file pone.0132148.s006.tif]
